# Reversal of Succinylcholine Induced Apnea with an Organophosphate Scavenging Recombinant Butyrylcholinesterase

**DOI:** 10.1371/journal.pone.0059159

**Published:** 2013-03-11

**Authors:** Brian C. Geyer, Katherine E. Larrimore, Jacquelyn Kilbourne, Latha Kannan, Tsafrir S. Mor

**Affiliations:** School of Life Sciences and The Biodesign Institute, Arizona State University, Tempe, Arizona, United States of America; TGen, United States of America

## Abstract

**Background:**

Concerns about the safety of paralytics such as succinylcholine to facilitate endotracheal intubation limit their use in prehospital and emergency department settings. The ability to rapidly reverse paralysis and restore respiratory drive would increase the safety margin of an agent, thus permitting the pursuit of alternative intubation strategies. In particular, patients who carry genetic or acquired deficiency of butyrylcholinesterase, the serum enzyme responsible for succinylcholine hydrolysis, are susceptible to succinylcholine-induced apnea, which manifests as paralysis, lasting hours beyond the normally brief half-life of succinylcholine. We hypothesized that intravenous administration of plant-derived recombinant BChE, which also prevents mortality in nerve agent poisoning, would rapidly reverse the effects of succinylcholine.

**Methods:**

Recombinant butyrylcholinesterase was produced in transgenic plants and purified. Further analysis involved murine and guinea pig models of succinylcholine toxicity. Animals were treated with lethal and sublethal doses of succinylcholine followed by administration of butyrylcholinesterase or vehicle. In both animal models vital signs and overall survival at specified intervals post succinylcholine administration were assessed.

**Results:**

Purified plant-derived recombinant human butyrylcholinesterase can hydrolyze succinylcholine *in vitro*. Challenge of mice with an LD_100_ of succinylcholine followed by BChE administration resulted in complete prevention of respiratory inhibition and concomitant mortality. Furthermore, experiments in symptomatic guinea pigs demonstrated extremely rapid succinylcholine detoxification with complete amelioration of symptoms and no apparent complications.

**Conclusions:**

Recombinant plant-derived butyrylcholinesterase was capable of counteracting and reversing apnea in two complementary models of lethal succinylcholine toxicity, completely preventing mortality. This study of a protein antidote validates the feasibility of protection and treatment of overdose from succinylcholine as well as other biologically active butyrylcholinesterase substrates.

## Introduction

Rapid sequence intubation (RSI) combines a sedative and a neuromuscular blocking agent (NMBA) to prepare a critically ill patient for emergent intubation [1], [2]. RSI is considered a safe procedure in the well-controlled environment of the emergency department where a large, diverse and highly trained team is available to provide emergent intubation, and where increased safety is bolstered by the availability of a large variety of rescue devices [3]. In the pre-hospital arena, the role of RSI remains, however, controversial [4], [5], [6], [7]. While some studies have demonstrated benefit, particularly in specific groups such as head injury patients, other authorities stress the complexity of this procedure, the risks associated with inadequately qualified, trained and equipped personnel and the overall lack of large-scale risk-benefit validation [5], [6], [7], [8], [9]. The controversy is well-reflected in the official policy statement of the American College of Emergency Physicians (ACEP), which declares that it “is not an advocacy statement for or against the use of” RSI [8].

Furthermore, disagreement exists over the ideal NMBA, but frequently centers on the non-depolarizing NMBA rocuronium and the depolarizing NMBA succinylcholine (SC, suxamethonium)[10], [11]. Of the two, consensus generally leans towards succinylcholine due to increased control afforded by the much shorter half-life, unless a contraindication exists [12], [13], [14]. Obvious contraindications include hyperkalemia, allergy, history of malignant hyperthermia, denervation syndromes or recent burn or crush injury. A more occult contraindication is delayed metabolism through the genetic absence of the SC hydrolyzing enzyme butyrylcholinesterase (BChE, plasma cholinesterase, pseudocholinesterase)[15].

This condition, succinylcholine apnea, is believed to exist in 1 in 1800 administrations of SC[16]. Of these, approximately 65% are caused by decreased SC hydrolysis by BChE variants with decreased hydrolytic function or decreased protein stability leading to lower effective serum levels[17]. Individuals who are homozygous in respect to the “atypical” (A) variant of the BChE gene (D70G, residual serum BChE activity is ∼30% of normal)[18], [19] experience periods of apnea of approximately 2 hours upon receiving an i.v. dose of SC that would otherwise result in only 3–5 minute-long paralysis in people with the common or “usual” (U) allele [17], [20]. Homozygocity toward several (∼48) other mutations in the BCHE locus lead to an even more drastic phenotype. These “silent” (S) variants of BChE and the affected patients are apneic for a much longer duration of 3–4 hours or even longer [17], [21]. While patients homozygous in respect to either the A or S variants are rare among the general population (∼0.03% and ∼0.01% respectively), heterozygotes are much more common (4% of the general population carry at least one A allele) and exhibit varying degrees of post-SA apnea depending on their specific combination of alleles and in direct correlation with their serum BChE activities [17].

The remaining cases of BChE deficiency are acquired through conditions decreasing circulating hepatoproteins such as cirrhosis [22], burns [23], [24], HELLP (hemolysis, elevated liver enzymes and low platelet count) syndrome [25], hepatic carcinoma [26] and malnutrition [27]. Furthermore, functional BChE deficiency can be acquired through the use of commonly prescribed drugs affecting production or function of BChE such as oral contraceptives [28], sertraline[29], cyclophosphamide[30], [31], [32], tacrine[33], phenelzine[34], bambuterol[35], metoclopramide[36], and ecothiophate[37], [38]. Beyond the rare case of genetic or acquired SC apnea, the ability to rapidly reverse the effects of SC could provide a significantly increased safety margin for the agent by allowing the return of spontaneous respirations and the pursuit of other management strategies including alternative medications and airway devices. This is particularly true in cases of upper airway obstruction where neuromuscular blockade carries the risk of airway collapse [39].

Current management approaches for post-SC apnea, a typically self-limiting condition, are supportive and highly resource intensive[40], mainly because of the lack of effective reversal agents. Anticholinesterases such as edrophonium and neostigmine, presumably intended to overcome cholinergic blockade by raising synaptic levels of acetylcholine, have been tested with underwhelming results [41]. The missing or nonfunctional serum BChE can be replaced by the active U-variant of the enzyme present in blood products like stabilized serum [42], fresh frozen plasma [43] or the purified enzyme (previously available for human use in Europe, but currently discontinued) [44] but the treatment carries the usual risks of blood-borne pathogens and prions, as well as the more common transfusion associated complications, including transfusion-related acute lung injury (TRALI)[45].

To take advantage of recent advances in biotechnology, we and others have hypothesized that purified recombinant human BChE would serve as an ideal antidote for SC apnea by avoiding risk of infection, TRALI and supply limitations [46], [47], [48]. Our group has previously demonstrated, in both mice and guinea pigs, the efficacy of recombinant BChE and acetylcholinesterase prophylaxis to prevent morbidity and mortality in organophosphate poisoning [49], [50]. These two animal species offer the prediction power expected of an animal model with small body sizes that limit the quantities of test materials required and ease of physiological measurements. Here we report SC hydrolysis and reversal of apnea and cardiovascular collapse with treatment of affected mice and guinea pigs by catalytic quantities of BChE.

## Materials and Methods

### Preparation of recombinant butyrylcholinesterase

Transgenic plants expressing a plant-optimized synthetic gene encoding full-length wild-type human BChE were created as previously described [51]. Briefly, stable *Nicotiana benthamiana* lines expressing a codon-optimized human butyrylcholinesterase were created and screened for maximal expression. The lines with highest accumulation were expanded from homozygous seed stocks and propagated under greenhouse conditions. Plant-derived BChE (pBChE) was prepared from mature 8–11 week old plants that were juiced in the presence of 150 mM sodium metabisulfite, and the juice was strained and clarified by centrifugation. The 30%–70% ammonium sulfate fraction (pH 4.0) was resuspended and subjected to two affinity chromatography steps, first through Concanavalin A-Sepharose 4B and then procainamide-agarose gel custom resin. Eluate was dialyzed against 0.125× phosphate-buffered saline (PBS), pH 7.4, then concentrated and stored with 0.02% azide at 4°C for up to 6 months. Prior to use, the preparation was dialyzed again against.125× PBS to remove azide. As previously described, our preparations of plant-derived BChE contain mostly tetramers (about a half) and monomers (about a third) [50].

### Biochemical analysis

Assay of butyrylthiocholine hydrolysis followed the method of Ellman as described in [51]. Succinylcholine hydrolase activity was monitored by the method of George and co-workers [52] with modifications to fit a 96-well plate format. Briefly, our standard succinylcholine-hydrolysis buffer contained 100 mM NaH_2_PO_4_/Na_2_HPO_4_ buffer pH 7.5, 0.77 mM phenol, 0.15 mM 4-aminoantipyrine, 1 U/mL choline oxidase, and 1.2 U/mL horse radish peroxidase type I. Appropriate volumes of 10× stock solutions (in 100 mM NaH_2_PO_4_/Na_2_HPO_4_ buffer pH 7.5) were pre-mixed and dispensed at 160 µL aliquots onto 96-well plates followed by addition of the substrate succinylcholine chloride (20 µl, final concentrations as indicated). Reactions were started by addition of pBChE (4.74 nM) to yield a final well volume of 200 µl. Hydrolysis was monitored by recording absorbance changes at 500 nm. Self hydrolysis rates were measured on samples that contained no enzyme and were subtracted from the enzymatically catalyzed reaction rates. A choline standard curve was similarly created by using the same assay except that choline chloride replaced succinylcholine (final concentration range of 10–100 µM).

Kinetic analysis was done according to Radić et al. [53] as follows. Initial enzyme velocity, V_0_, was plotted as a function of substrate concentration and the results were fitted by nonlinear regression using GraphPad Prism to the following equation:

where [SC] is the concentration of SC, V_max_ is maximal velocity, K_M_ is the Michaelis-Menten constant, K_SS_ is the dissociation constant of substrate from the enzyme's peripheral binding site, and *b* is a factor that reflects the efficiency of hydrolysis of the substrate in the presence of another substrate molecule bound at the peripheral site (with substrate activation when b>1).

### *In vivo* experiments

All animal experiments were conducted in accordance to specific protocols approved by the Institutional Care and Use Committee of Arizona State University (approval number 07-911R).

Mouse experiments were conducted as follows. Male FVB/N mice (*Mus musculus*, 8–12 weeks old) were anesthetized by injection of ketamine/xylazine/acepromazide cocktail at the dose of 0.05 mL per 25 g of total body weight (concentrations were, respectively 21 mg/mL, 2.4 mg/mL, and 0.3 mg/mL). Anesthetized mice were assessed for respiratory rate by counting respirations for 30 seconds using a Littman model 3000 electronic stethoscope placed on the mouse left mid axillary line and extrapolating the per minute rate. Mice were then injected intravenously (tail vein) with 1 mg/kg succinylcholine (time 0 min). At time +3 min mice were injected with pBChE (0.6 mg/kg, ∼11 U per animal, *n* = 3) in 0.9% saline (or 0.9% saline vehicle control, *n* = 3) and respiratory rate was obtained as above at the indicated time points. At time +15 minutes all surviving mice were euthanized by CO_2_ asphyxiation and subsequent cervical dislocation. At no time during the experiment did the mice receive any other therapy including, but not limited to, airway protection/management, artificial ventilations, compressions, or any pharmacological assistance.

Guinea pig experiments were conducted as follows. Male Hartley guinea pigs (*Cavia porcellus*, 8 weeks old) were anesthetized with 90 mg/kg ketamine and 10 mg/kg xylazine. Once anesthetized, baseline heart rate (beats per minute) and SpO_2_ (%) were obtained using a Surgivet Plus Veterinary Anesthesia and Monitoring Module, model #V3404. Guinea pigs (3 per group) were then intravenously (leg vein) injected at t = 0 with SC at either low dose (0.167 mg/kg, experiment I) or a high dose (0.334 mg/kg, experiment II). Heart rate and SpO_2_ were obtained every minute throughout the course of the experiment. At time +1 min, groups of three apneic guinea pigs were injected with either pBChE in 0.9% saline at a low dose (0.09 mg/kg, ∼24 U per animal, experiment I), high dose (0.19 mg/kg, ∼48 U per animal, experiment II), or 0.9% saline vehicle control (experiment I and II). At t = +15 minutes, all surviving guinea pigs were euthanized by CO_2_ asphyxiation and subsequent cervical dislocation. As above, guinea pigs received no additional care or therapy during experimentation.

### Statistical analyses

Statistical analyses were carried out using the GraphPad Prism software. Log-rank (Mantel-Cox) test was used to determine significance of the difference between survival curves. Comparisons between mean values of heart rate and Sp0_2_ were tested using 1-way analysis of variance (ANOVA) followed by Bonferroni's Multiple Comparison Test. Results were also analyzed by 2-way ANOVA (testing simultaneous effects of time and BChE treatment) and simple t-tests, and the inferences based on these analyses were very similar to those obtained by the 1-way ANOVA analysis described above.

## Results

**Figure 1:**
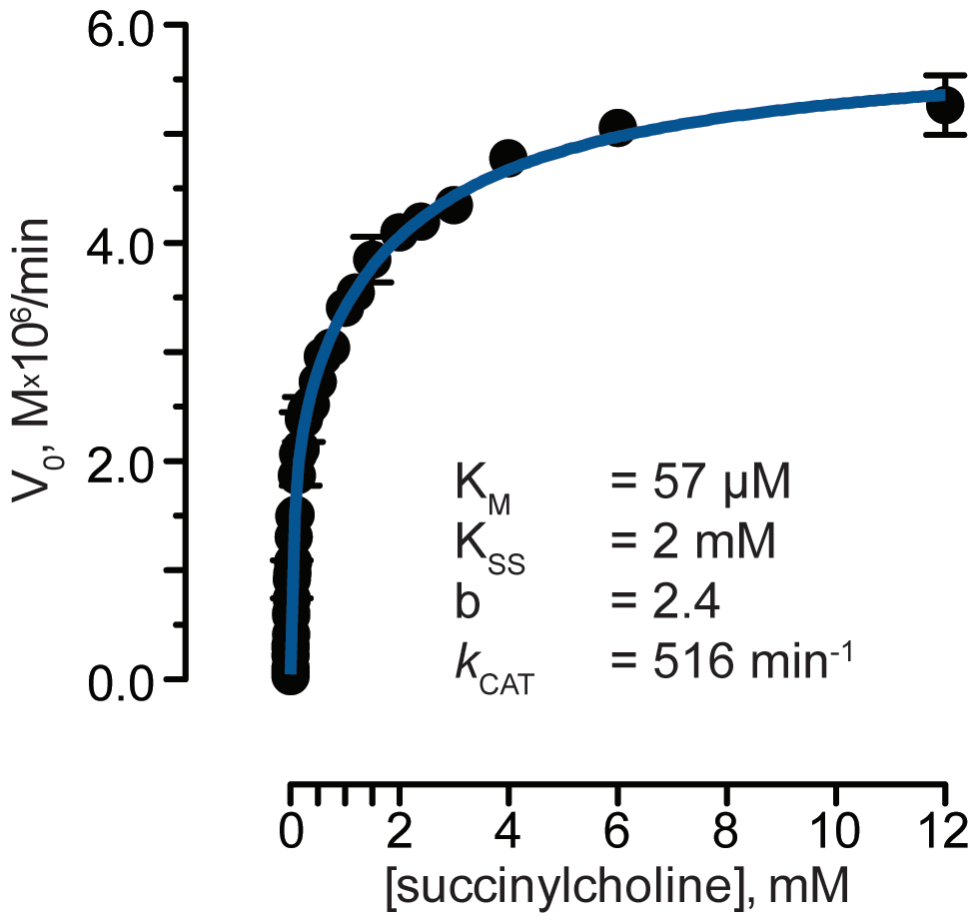
Plant-derived BChE undergoes substrate activation by its succinylcholine substrate. Blue line represents data fitted by nonlinear regression to fit the equation: 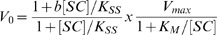
with the following parameters (±SEM): V_max_ = 2.45±0.16 µM/min, K_M_ = 57±7 µM, K_ss_ = 2.0±0.3 mM, and b = 2.9±0.1. *k*_cat_ = V_max_/[BChE]_T_ was calculated to be 516±33 min^−1^ based on the above V_max_ value and [BChE] = 4.74 nM.

Initial characterization of plant-derived, recombinant human butyrylcholinesterase (pBChE) was previously published and was found to be indistinguishable from that of the human plasma-derived enzyme in its ability to interact with its acetylcholine and butyrylcholine substrates and various inhibitors [50], [51], [54]. These studies included detailed *in-vitro* and *in-vivo* demonstration of the ability of the plant-derived enzyme to scavenge organophosphate pesticides and nerve-agents (“nerve gasses”). Here we extend these studies in order to investigate the potential of pBChE to reverse SC-induced apnea and therefore determined its SC hydrolytic capacity. Succinylcholine hydrolysis by pBChE proceeded in a linear time-dependent manner with BChE (data not shown) allowing us to calculate the initial enzyme velocity (V_0_). Conducting the experiment at increasing SC concentrations and plotting the V_0_ values as a function of the SC concentration (Fig. 1) allowed us to obtain the Michaelis constant (K_M_ = 57±7 µM) and the turnover number (*k*_cat_ = 516±33 min^−1^, Fig. 1). The catalytic efficiency (*k*_cat_/K_M_) was calculated to be 9×10^6^ M^−1^min^−1^. These values were consistent with previously published results for human BChE (K_M_ = 35 µM and *k*_cat_ = 600 min^−1^)[55]. Differences might be attributed to the difference in the assay used in the published research that employed an SA thioester analog. As is the case with many other substrates of BChE, hydrolysis of SC by the enzyme was shown to exhibit substrate activation, presumably due to allosteric interactions involving the peripheral substrate binding site.

**Figure 2:**
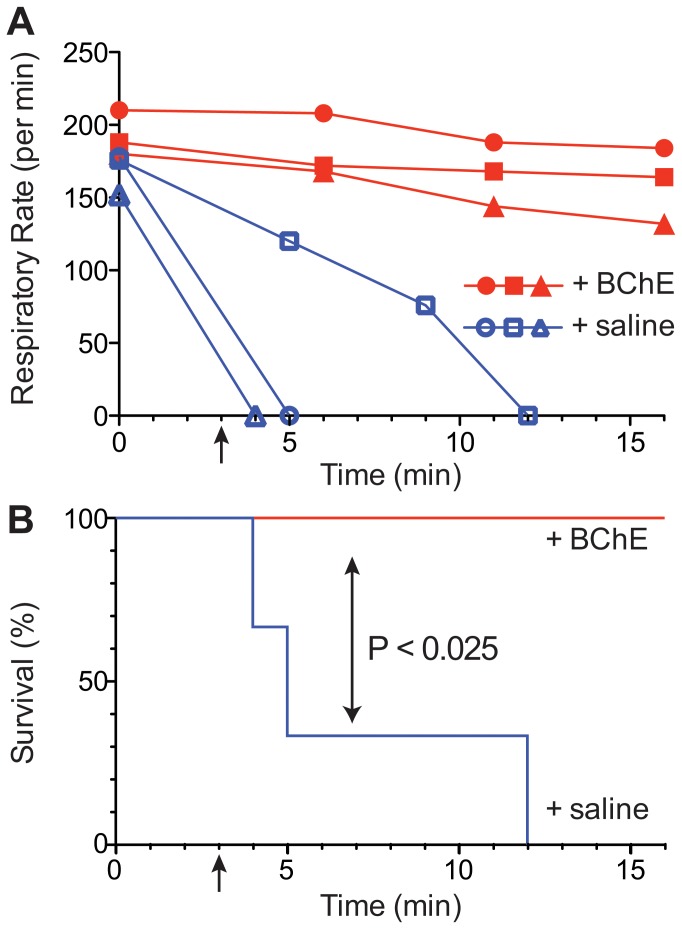
Plant-derived BChE protects mice from SC-induced apnea. A Respiration rate of mice treated with SC followed by administration of pBChE or saline was monitored. Symbols represent individual animal subjects (*n* = 3 for each group). B The Kaplan–Meier estimates of survival reflecting survival of all pBChE-treated mice as opposed to 100% mortality among control, saline-treated subjects. Comparison by the Log-rank (Mantel-Cox) test was significant (*P*<0.025).

To test our study's hypothesis that pBChE could reverse succinylcholine-induced apnea, we turned to animal-models in two species. Initial studies were conducted with mice that were administered intravenous SC, 1 mg/kg, a dose which constituted about >3× LD_50_ (0.28 mg/kg) [56] and in our hands proved to be lethal to 100% of tested animals. Mice were then randomized to receive either 15 U BChE or vehicle control (0.9% saline) at 3 min following SC injection. Respiratory rate was monitored every five minutes following SC injection. While all three mice receiving SC +0.9% saline succumbed to the SC-induced respiratory depression and subsequently died, all three mice receiving SC+15 U BChE survived (Fig. 2).

**Figure 3:**
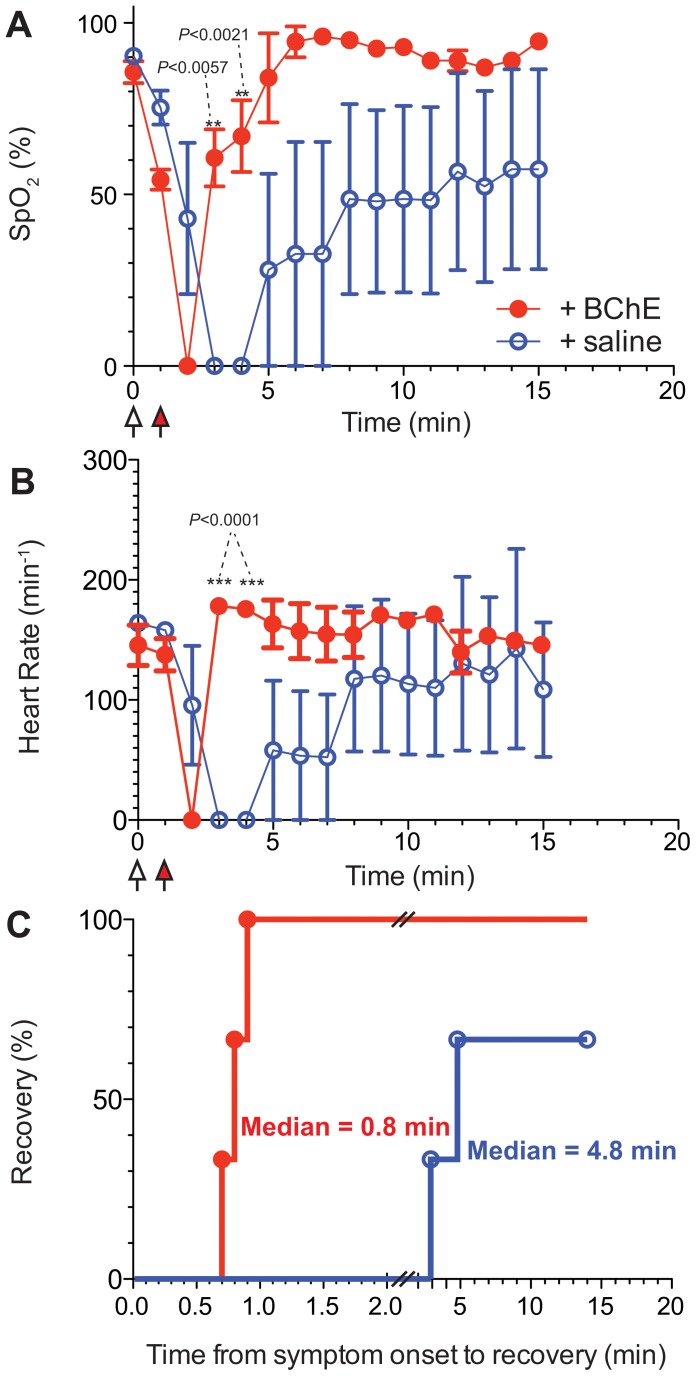
Plant-derived BChE facilitate recovery of guinea pigs treated with sublethal dose of SC. Guinea pigs were injected with SC (0.167 mg/kg) followed by injection with BChE (*n* = 3, red) or saline (*n* = 3, blue) while monitoring oxygen saturation (A) and heart rate (B). Reported data points represent means±SEM. Means at each time point were compared by 2-way repeated ANOVA and P values are denoted on the graphs. Statistical analyses by t-test or 1-way ANOVA yielded very similar results. C Time to return to normal heart rate. Comparison by the Log-rank (Mantel-Cox) test was significant (*P*<0.025).

**Figure 4:**
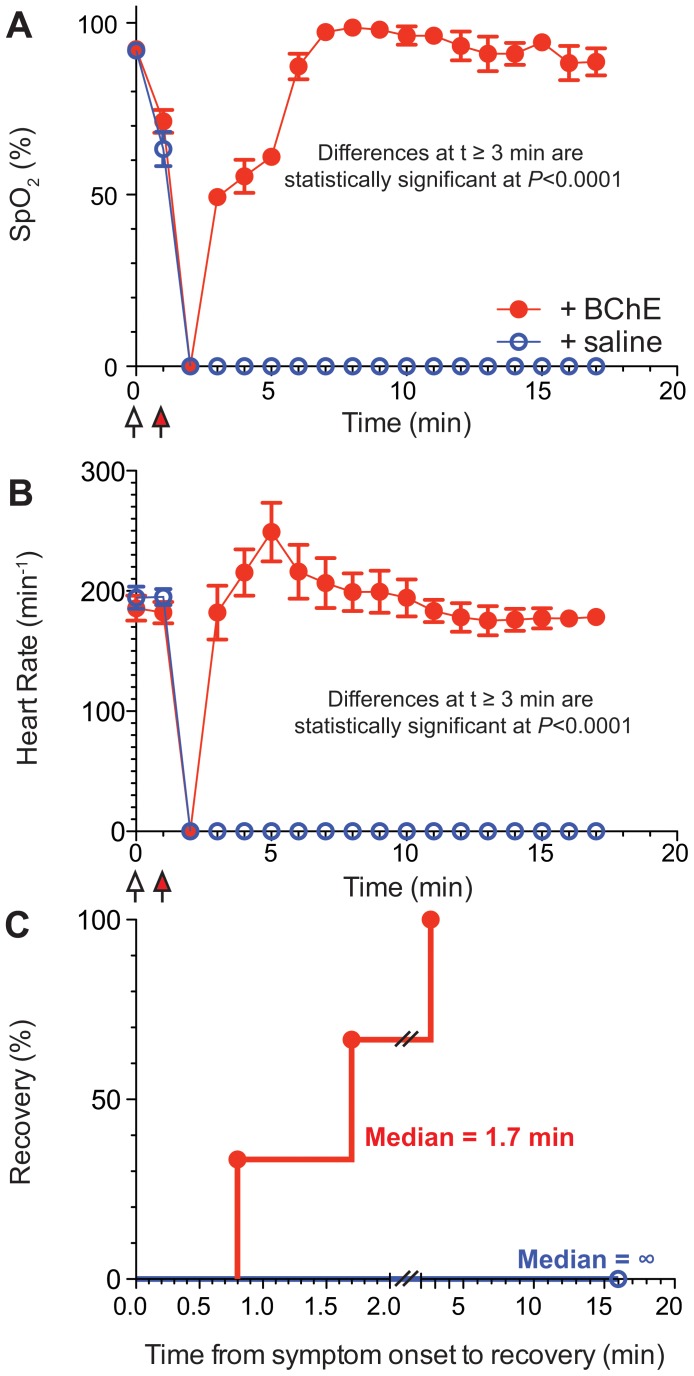
Plant-derived BChE fully protects guinea pigs from high-dose SC-induced apnea. Guinea pigs were injected with SC (0.334 mg/kg) followed by injection with BChE (*n* = 3, red) or saline (*n* = 3, blue) while monitoring oxygen saturation (A) and heart rate (B). Reported data points represent means±SEM. Means at each time point were compared by 2-way repeated ANOVA and P values are denoted on the graphs. Statistical analyses by t-test or 1-way ANOVA yielded very similar results. C Time to return to normal heart rate. Comparison by the Log-rank (Mantel-Cox) test was significant (*P*<0.025).

To further understand the effect and kinetics of BChE-mediated SC detoxification using continuous vital sign monitoring, we moved to a larger rodent model of SC toxicity. Utilizing guinea pigs, we intravenously injected groups of three animals with either 0.167 mg/kg (∼LD_50_, Fig. 3) or a larger dose that leads to 100% mortality (0.334 mg/kg, Fig. 4). Complete apnea and resultant decreases in oxygen saturation were seen in both groups at 1 min following SC injection, at which time study animals received either 24 U (Fig. 3), 48 U BChE or vehicle control (0.9% saline, Fig. 3 and Fig. 4). Animals in all four groups went on to demonstrate an absence of measurable pulse and oxygen saturation within 2 to 3 min following SC injection (Fig. 3 and 4). Within 2 minutes, guinea pigs receiving BChE recovered spontaneous respirations, their venous oxygenation level had risen to about 50% and their heart rate was at baseline. In fact, median time to recovery was 0.8 min for animals treated with the low dose of SC and 1.7 min for the high dose of SC. We witnessed a fairly consistent response of post-anoxic tachycardia in recovering guinea pigs at 5 min post SC injection, however by 7 min post SC injection all vital signs had returned to baseline in all animals treated by BChE. In striking contrast, only two of the three saline-treated control animals survived with a median time to recovery of 4.8 min. Thus, control animals experienced slower recovery (median ratio saline/BChE = 6.0, 95% confidence interval of 5.8 to 6.2) following a low-dose SC exposure. In this case the hazard ratio of BChE-treated and controls was 15.34 (95% confidence interval of 1.418 to 166.0). Moreover, all animals exposed to high dose of SC without BChE treatment died without any recovery of viable signs. At no point following loss of measurable pulse and venous oxygen saturation did these signs return in animals receiving the high dose of SC without the treatment +0.9% saline.

## Discussion

This study demonstrates the capability of plant-derived recombinant human BChE to completely reverse the effects of SC paralysis and respiratory compromise in two small animal models where no additional supportive care was provided. Our work provides a natural extension to previous work that demonstrated the proof of principle for enzyme replacement therapy to counteract the effects of profound neuromuscular SC blockade in patients with BChE deficiencies [42], [43], [44]. All of these previous reports centered around the human plasma enzyme, as present in various blood products or purified from the latter. Plasma-derived BChE is expensive, exists in greatly limited supply, and is, consequently, of limited utility. Our work demonstrates, for the first time, the application of recombinant BChE produced in a sustainable fashion in plants as an antidote to SC-induced neuromuscular blockade. Plant-based production platforms as a source for protein pharmaceuticals are only now beginning to be tapped into with a recent approval of carrot-cell derived glucocerebrosidase for the treatment of Gaucher disease [57], [58]. Our results provide, in two small animal models, the proof of principle that SC-induced apnea can be reversed by administration of plant-produced recombinant human BChE. While our studies were conducted in small number of animals, they provide evidence that is statistically significant, for full protection afforded by the plant-produced enzyme. Additional future tests will aim to look at more subtle biological outcomes (e.g. immune responses to the plant-derived biologics), to determine the safety and efficacy and are likely to require larger number of subjects per group to yield the necessary power.

The ability to rapidly reverse the effects of SC may have profound clinical implications in cases ranging from genetic or acquired SC apnea to SC overdose and particularly traditional RSI utilizing SC as the paralytic. Failed airways are a relatively common event in both the emergency department and the prehospital arena and the decision to adopt an alternate strategy may be hampered by lingering paralysis and the absence of spontaneous respirations. While RSI in emergency medical service is common in some areas, many other agencies have removed paralytics from the drug box of first responders over concerns of apnea, prolonged hypoxia and cardiovascular collapse. In addition to simulation, increased training intervals and close oversight, pharmacological reversal of SC paralysis may increase the safety margin associated with RSI in prehospital medicine.

In broad terms, the search for antidotes must balance efficacy with practicality. While recombinant proteins offer the potential for rational design and mutation of naturally occurring molecules, the increased cost and relatively decreased shelf life encourages exploration of alternate uses for these technologies so they can be applied toward multiple indications. Treatment of SC-induced apnea offers an alternative use for recombinant BChE, beyond scavenging of organophosphate nerve agents in a chemical warfare/terrorism scenario [47], [50], [51], that may provide increased patient safety in the use of RSI in both hospital and pre-hospital care. Moreover, SC is used in clinical contexts beyond RSI, for example in conjunction with electroconvulsive therapy, where BChE variants may have a negative impact as was recently shown by Mollerup and Gätke [59]. Further potential use of BChE includes rapid cocaine detoxification in overdose as well as pharmacological protection of cocaine “packers” during GI clearance [60], [61], [62].

Determination of the appropriate agent and venue for RSI must balance safety and efficacy with continuous measurement of patient outcomes. The availability of plant-derived BChE to rapidly clear SC and reverse apnea should add a new facet to the debate over the ideal paralytic and potentially prevent some of the more common outcomes of prolonged hypoxia during the management of difficult airways using SC [10], [11], [12], [13], [14]. Beyond the rare case of genetic or acquired SC apnea, the ability to rapidly reverse the effects of SC could provide a significantly increased safety margin for the agent by allowing the return of spontaneous respirations and the pursuit of other management strategies including alternative drugs and airway devices. While we acknowledge that a reversal drug to remove excess SC leading to neuromuscular blockade is only one aspect of a much more complicated clinical scenario, we hope that the preclinical work presented would revive interest in testing plant-derived BChE as a tool to counteract SC-induced paralysis in a clinical setting. Such a clinical trial will have to take place following Phase I trials to demonstrate safety of this protein drug. Work at our lab continues toward this goal.

## Conclusions

In two complementary small animal models of succinylcholine-induced apnea, recombinant butyrylcholinesterase reversed apnea, desaturation and loss of pulse and prevented mortality. Use of BChE may provide an additional tool to increase the safety of succinylcholine in emergent intubation.
